# Nitric oxide mediates apoptosis and mitochondrial dysfunction and plays a role in growth hormone deficiency by nivalenol in GH3 cells

**DOI:** 10.1038/s41598-017-16908-y

**Published:** 2017-12-06

**Authors:** Deyu Huang, Luqing Cui, Pu Guo, Xijuan Xue, Qinghua Wu, Hafiz Iftikhar Hussain, Xu Wang, Zonghui Yuan

**Affiliations:** 1 The Key Laboratory for the Detection of Veterinary Drug Residues, Ministry of Agriculture, Wuhan, P.R. China; 2 Laboratory of Quality & Safety Risk Assessment for Livestock and Poultry Products (Wuhan), Ministry of Agriculture, Wuhan, P.R. China; 3 College of Life Science, Yangtze University, Jingzhou, Hubei 434025 P.R. China

## Abstract

Nivalenol (NIV), a type B trichothecenes commonly found in cereal crops, can cause growth impairment in animals. However, limited information about its mechanisms is available. Trichothecenes have been characterized as an inhibitor of protein synthesis and induce apoptosis in cells. Oxidative stress is considered an underlying mechanism. However, whether NIV can induce oxidative stress and apoptosis in rat pituitary cells line GH3 is unclear. The present study showed that NIV significantly reduced the viability of cells and caused oxidative stress in GH3 cells. Further experiments showed that nitric oxide (NO), but not ROS, mediated NIV-induced oxidative stress. Additionally, NIV induced caspase-dependent apoptosis, decrease in mitochondrial membrane potential and mitochondrial ultrastructural changes. However, NIV-induced caspase activation, mitochondrial damage and apoptosis were partially alleviated by Z-VAD-FMK or NO scavenger hemoglobin. Finally, NIV changed the expression of growth-associated genes and pro-inflammatory cytokines. NIV also reduced the GH secretion in GH3 cells, which was reversed by hemoglobin. Taken together, these results suggested that NIV induced apoptosis in caspase-dependent mitochondrial pathway in GH3 cells, which might be an underlying mechanism of NIV-induced GH deficiency. Importantly, NO played a critical role in the induction of oxidative stress, apoptosis and GH deficiency in NIV-treated GH3 cells.

## Introduction

Nivalenol (NIV), a type B trichothecenes mycotoxin, is commonly found as a contaminant in cereals like wheat, maize and barley^1,2^. In animals, NIV can induce a series of toxic actions including diarrhea, emesis, anemia and suppression of appetite^3^. This toxin also leads to tremendous economic losses due to reduced weight gain, less milk production and insufficient reproductive ability in animals^4^.

A very important toxicity for trichothecenes is growth retardation in food animals, which is an evidence for establishing the tolerated daily intake of 0.7 μg/kg b.w. for NIV by the World Health Organization Joint Expert Committee on Food Additives^5^. The regulation of growth is complex, involving a number of molecules like growth hormone (GH), growth hormone receptors (GHRs), insulin-like growth factor 1 (IGF-1), insulin-like growth factor acid-labile substance (IGF-ALS) and insulin-like growth factor binding protein-3 (IGFBP-3). Briefly, GH binds to hepatic GHRs, activating mitogen-activated protein kinases (MAPKs) and signal transducers and activators of transcription (STATs). The STATs translocate to the nucleus and upregulate expression of IGF-1, IGF-ALS and IGFBP-3^6^. Suppressors of cytokine signaling (SOCS) can negatively regulate this process at the level of GHRs^7^.

Trichothecenes have been characterized as an inhibitor of protein synthesis as they could bind to the 60 S ribosomal subunit and therefore activated the MAPKs signaling pathway. This process was termed as ribotoxic stress response^8^. However, so far, the exact mechanism regarding growth retardation induced by trichothecenes is not fully clear. The most studied trichothecenes relative to growth retardation is DON. It has been reported that DON suppressed growth in mice by reducing GH signaling through mechanisms mediated by IGF-1 and IGF-ALS^9^. DON could possibly act directly on the pituitary glands of rats to change the mode of pituitary hormone secretion and decreased the body weight gain^10^. Another study showed that NIV had a negative effect on body weight gain in F344 rats. Pathological changes were observed in the anterior pituitary including an increase of castration cells and development of diffuse hypertrophy of basophilic cells^11^. The findings of Wan *et al*.^12^ proved that T-2 toxin and DON inhibited *GH* gene and protein expression and decreased GH secretion in GH3 cells. Although it is known that NIV can decrease the weight gain in animals, the underlying mechanism is still unclear.

NO is an important oxidative biological molecule in a variety of physiological processes including neurotransmission, blood pressure regulation, smooth muscle relaxation and immune regulation^13^. The correlation between trichothecenes and NO was studied. It was found that DON and NIV suppressed the lipopolysaccharide (LPS)-induced NO production and transcriptional activation of inducible NO synthase (NOS) in RAW264 cells^14^. In LPS-treated murine dendritic cells, the involvement of NO in cell maturation process was downregulated by DON and NIV through reducing the NO production, and this could result in suppression of the immunological functions of the cells^15^. These studies suggest that NO have a role in the trichothecene-induced immunosuppression. However, as a free radical, NO could initiate the oxidative stress which caused lipid peroxidation, DNA oxidative damage and induced apoptosis in human gingival fibroblast^16^ and human cervix carcinoma cells^17^ through the mitochondria dependent pathway. However, the involvement of NO in NIV-induced apoptosis has rarely been studied.

Pro-inflammatory cytokine expression can negatively affect growth and weight gain^9^. Transgenic mice overexpressing the cytokine IL-6 since birth showed a marked decrease in growth rate and weight gain compared with the wild type. In these mice, the induction of GH was normal, while the level of IGF-1 was significantly decreased^18,19^. IL-6-deficient mice showed decreased body composition and developed mature-onset obesity, which were partly reversed by IL-6 replacement treatment^20^. Thus it is reasonable to postulate that upregulation of pro-inflammatory cytokines could be a factor in NIV-induced growth impairment of animals.

The present study aimed to explore the mechanisms of GH deficiency in GH3 cells by NIV. The cytotoxicity, oxidative stress, apoptosis, mitochondrial damages, inhibition of GH secretion and growth-associated genes in GH3 cells induced by NIV were investigated. Furthermore, the critical role of NO in activating the caspase signaling pathway, mitochondrial damages and GH secretion were also investigated. This study will give a hint for the therapy of NIV-induced growth impairment in animals.

## Results

### NIV-induced cytotoxic effects on rat pituitary GH3 cells

**Figure 1:**
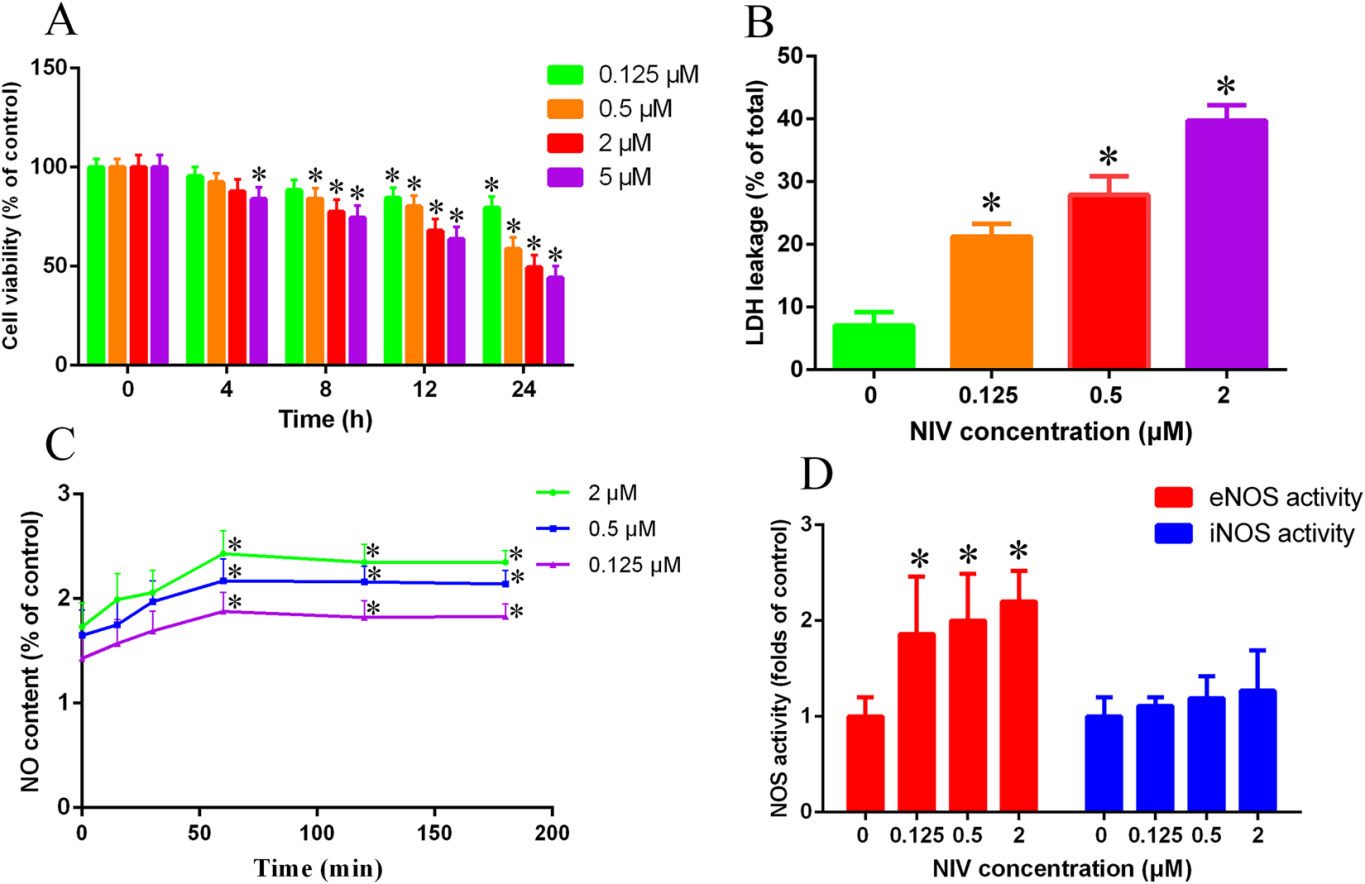
NIV induced cytotoxic effects in rat pituitary GH3 cells evaluated by MTT assay (**A**), LDH leakage assay (**B**), NO generation (**C**) and change of NOS activity (**D**). The LDH leakage was calculated as the percentage of LDH in the cell medium versus total LDH activity in the cells. NO was indicated by the fluorescence of the treated samples compared to the control samples. The results were expressed as mean ± SD of 3 separate experiments performed in triplicate and were statistically analyzed using one-way ANOVA. *p < 0.05 denotes statistical significance versus control.

The exposure time and concentrations in cells depended primarily on the detection of cytotoxicity, such as MTT assay and LDH leaking assay^21,22^. In the present study, the results of MTT assay displayed a concentration- and time-dependent decrease in cell viability induced by NIV in rat pituitary GH3 cells (Fig. 1A). The cells viability decreased to 84.52%, 80.13%, 67.93% and 63.74% after 12 h and to 79.50%, 58.68%, 49.41% and 44.13% after 24 h, at 0.125, 0.5, 2 and 5 μM concentration of NIV, respectively. The IC50 of NIV at 12 h to GH3 cells was calculated to be 25.00 μM. After 12 h of incubation, 0.125, 0.5 and 2 μM NIV resulted in 21.24%, 27.88% and 39.75% LDH leakage (% of total), which were statistically significant compared to the control (p < 0.05) (Fig. 1B). Though the IC50 of NIV at 12 h was 25.00 μM, while considering the IC50 results from MTT and LDH leakage as well as the state of GH3 cells, the concentrations of 0.125 μM, 0.5 μM and 2 μM of NIV and 12 h of exposure time were selected for the subsequent experiments.

### Effects of NIV on intracellular NO and NOS activity

The generation of NO and NOS activity were investigated after NIV treatment at 0.125, 0.5 and 2 μM for 12 h. NO level significantly increased and lasted as long as 3 h, reaching its peak level (1.8~2.4 fold) in about 60 minutes (Fig. 1C). In order to demonstrate the role of NOS in NO production, the NOS activity was measured (Fig. 1D). The results showed that NIV increased the endothelial NOS activity but not the inducible NOS activity to increase NO release in GH3 cells. These results suggested that NO might be a key mediator of NIV-induced cell death in rat pituitary GH3 cells.

### Effects of NIV on oxidative stress

**Figure 2:**
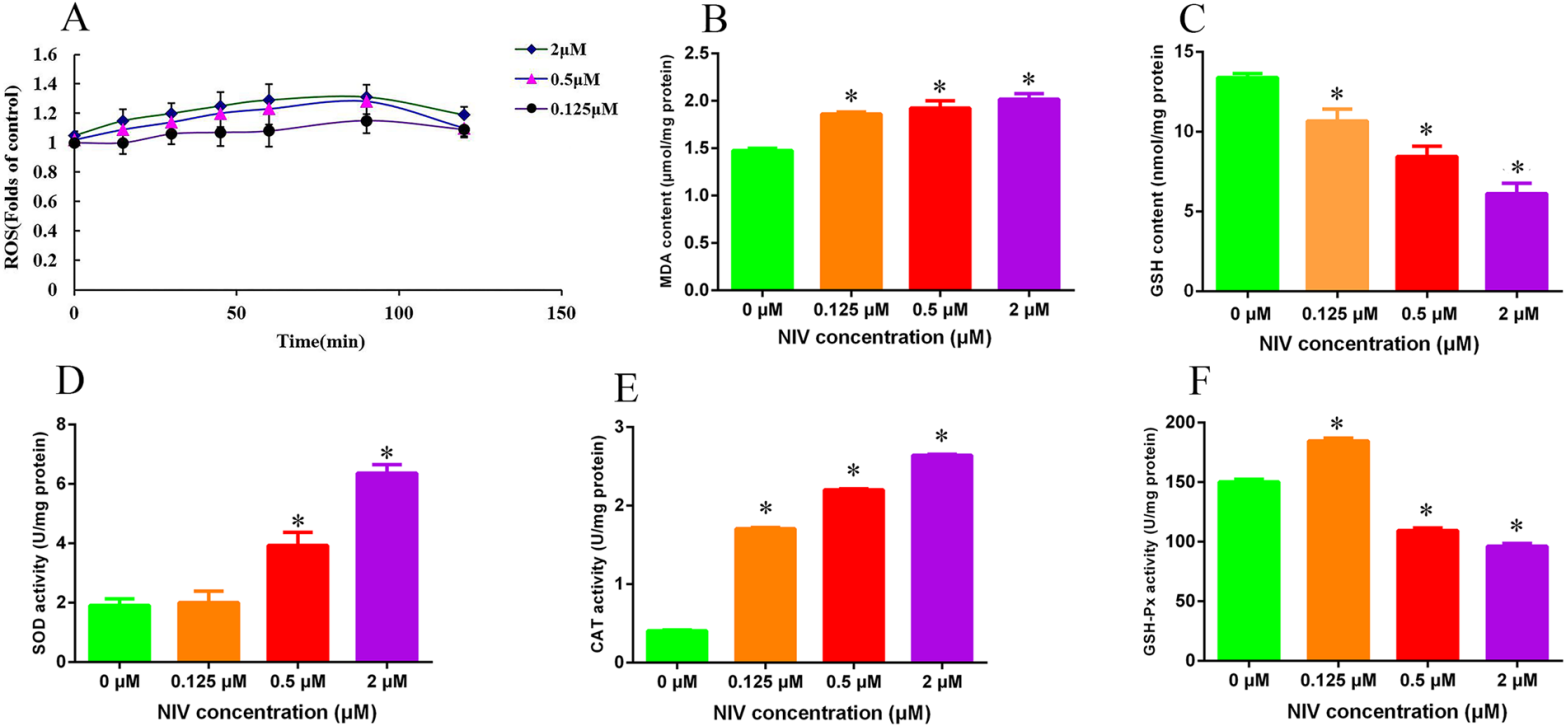
NIV-induced oxidative stress indicated by ROS level (**A**), MDA level (**B**), GSH (**C**), SOD (**D**), CAT (**E**), GSH-Px (**F**) activity in GH3 cells. ROS was indicated by the fluorescence of the treated samples compared to the control samples and was expressed as mean ± SD of 3 separate experiments performed in triplicate. The results of MDA level, GSH level, SOD, CAT and GSH-Px activity were expressed as mean ± SD of 6 separate experiments performed in triplicate. All data were statistically analyzed using one-way ANOVA. *p < 0.05 denotes statistical significance versus control.

Oxidative stress is involved in the toxicities of NIV in GH3 cells. In the present study, oxidative stress was evaluated in terms of reactive oxygen species (ROS), malonyldialdehyde (MDA), glutathione (GSH) and the activity of antioxidant enzymes superoxide dismutase (SOD), catalase (CAT) and glutathione peroxidase (GSH-Px). NIV did not significantly increased the ROS level although there was an ascending trend for it (p > 0.05) (Fig. 2A). However, NIV significantly increased the MDA level (p < 0.05) (Fig. 2B) and decreased the GSH level (p < 0.05) (Fig. 2C). NIV also significantly increased the activity of SOD (0.5 and 2 μM) (p < 0.05) (Fig. 2D) and the activity of CAT (0.125, 0.5, 2 μM) (p < 0.05) (Fig. 2E). Interestingly, NIV at 0.125 μM significantly increased the GSH-Px activity but significantly decreased it at 0.5 μM and 2 μM (p < 0.05) (Fig. 2F). The results suggested an imbalance of redox homeostasis in GH3 cells.

### Induction of apoptosis by NIV in GH3 cells

**Figure 3:**
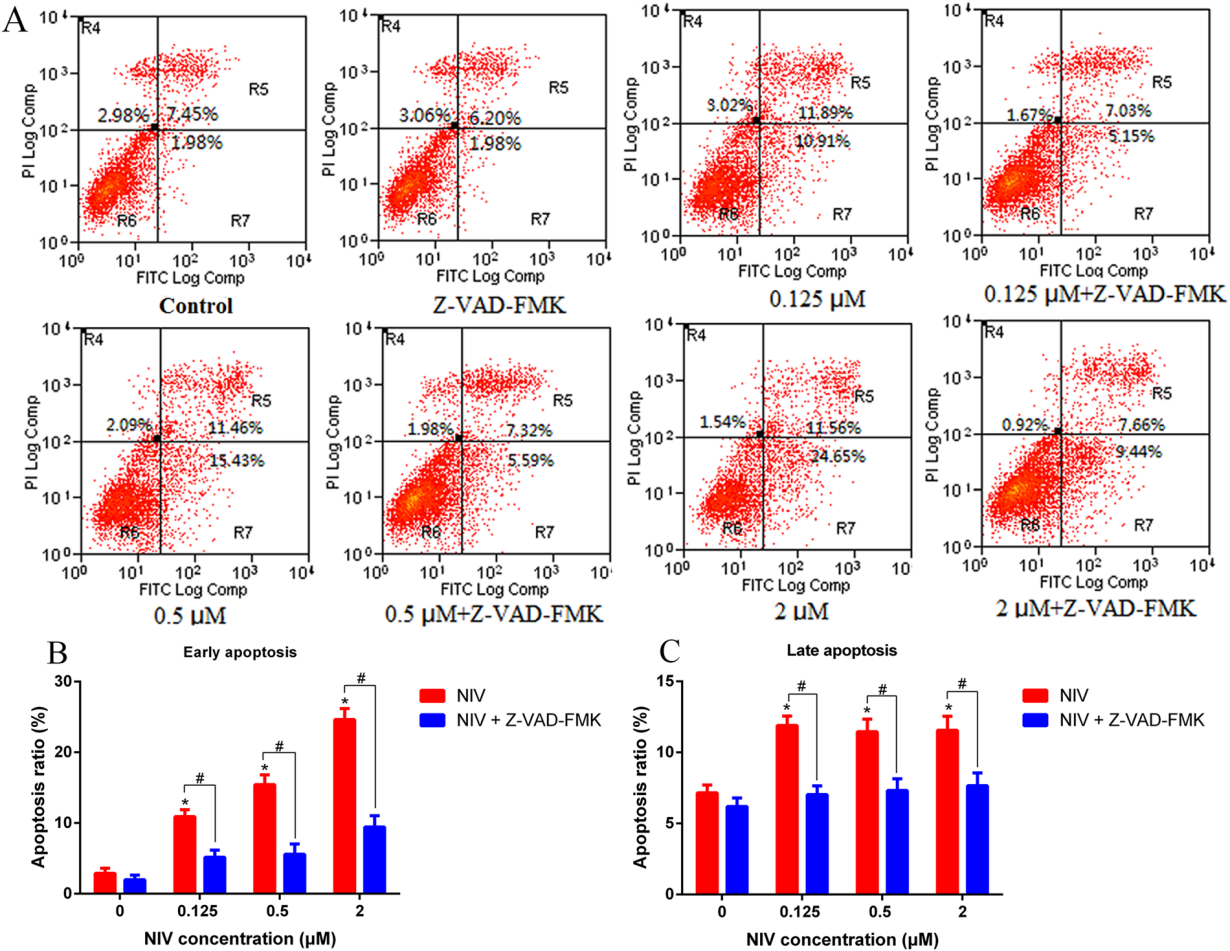
NIV induced apoptosis in GH3 cells. NIV increased the apoptosis (**A**), including the early (**B**) and late (**C**) apoptosis. The results were expressed as mean ± SD. n = 3 independent experiments. Comparisons between multiple groups with different concentrations of NIV (0.125, 0.5 and 2 μM) were analyzed using a one-way ANOVA. *p < 0.05 denotes statistical significance versus control. Comparisons between two groups with or without Z-VAD-FMK or hemoglobin were analyzed using Student’s t-test. ^#^p < 0.05 denotes statistical significance versus NIV alone treated cells. Note: R4 phase: necrotic cells; R5 phase: late apoptosis cells; R6 phase: live cells; R7 phase: early apoptosis cells.

By using an Annexin V-FITC/PI apoptosis detection kit with flow cytometry, it was revealed that NIV induced apoptosis in GH3 cells (Fig. 3A). A significant increase in the number of early apoptotic cells (R7 phase) as well as late apoptotic cells (R5 phase) was observed in NIV treated cells compared to the control (Fig. 3B,C). The number of early apoptotic cells, but not the late apoptotic cells, increased in a concentration-dependent manner. However, pretreatment of cells with caspase inhibitor Z-VAD-FMK significantly reduced the apoptotic effect of NIV in GH3 cells.

### Activation of caspase signaling pathway by NIV in NO-dependent manner

**Figure 4:**
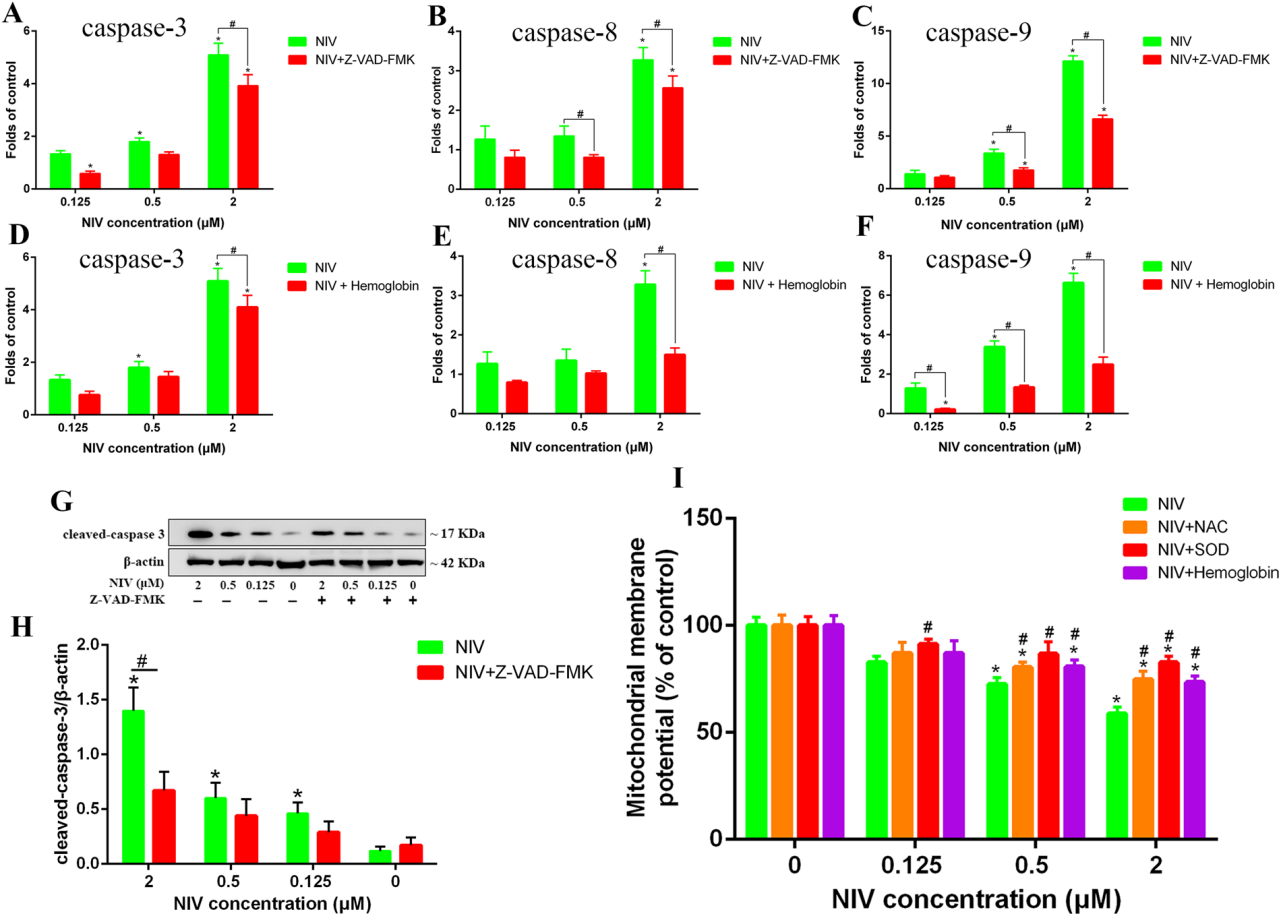
NIV-induced caspase activation and the effect of NIV on ΔΨm. The gene expression of caspase-3 (**A**,**D**), caspase-8 (**B**,**E**) and caspase-9 (**C**,**F**) induced by NIV without or with Z-VAD-FMK (**A**,**B**,**C**) or hemoglogin (**D**,**E**,**F**) was assessed by qRT-PCR. The data was expressed as mean ± SD of 3 separate experiments performed in triplicate. The protein level of cleaved-caspase-3 induced by NIV without or with Z-VAD-FMK was assessed by western blot (**G**) and quantified and presented in graph (**H**). NIV-induced decrease of ΔΨm without or with pretreatment of NAC, SOD and hemoglobin was measured (**I**). Comparisons between multiple groups with different concentrations of NIV (0.125, 0.5 and 2 μM) were analyzed using a one-way ANOVA. *p < 0.05 denotes statistical significance versus control. Comparisons between two groups without or with Z-VAD-FMK, hemoglobin, NAC or SOD were analyzed using Student’s t-test. ^#^p < 0.05 denotes statistical significance versus NIV alone treated cells.

The effect of NIV and Z-VAD-FMK on mRNA level of caspase-3, -8 and -9 was also measured. As we can see in Fig. 4, NIV activated the caspase signaling pathway by increasing the mRNA level of caspase-3, -8 and -9 (Fig. 4A,B and C). NIV (0.5 μM) significantly increased the mRNA level of caspase-3 and caspase-9 (p < 0.05). NIV (2 μM) also increased the mRNA level of caspase-3, -8 and -9 with a higher-fold change (p < 0.05). However, the increase of mRNA level of caspase-3, -8 and -9 induced by NIV was alleviated by Z-VAD-FMK, proving that NIV-induced apoptosis of GH3 cells is caspase-dependent.

NO is an important signal molecule in physiological processes. As shown in Fig. 4D,E and F, NO mediated the activation of the caspase signaling pathway induced by NIV. The NO scavenger hemoglobin (20 μM) abated NIV-induced activation of the caspase signaling pathway by decreasing the mRNA level of caspase-3, -8 and -9.

Given that caspase 3 protease is the most important end-cleaving protease in the process of apoptosis, the protein level of cleaved-caspase-3 was detected. As we can see, NIV at all concentrations of 0.125, 0.5 and 2 μM significantly increased the expression of cleaved-caspase-3 (Fig. 4G,H). However, Z-VAD-FMK significantly decreased the expression of cleaved-caspase-3 induced by 2 μM NIV, which indicated that caspase signaling pathway played a critical role in NIV-induced apoptosis of GH3 cells.

### Effects of NIV on mitochondrial membrane potential (ΔΨm)

As shown in Fig. 4I, after treatment of NIV at 0.125, 0.5 and 2 μM, the ΔΨm decreased to 82.72%, 72.52% and 58.87%, respectively. However, two free radical scavengers, NAC and SOD, and one NO scavenger, hemoglobin, all partially reversed the decrease of ΔΨm induced by NIV, indicating that oxidative stress and NO were involved in mitochondrial damage induced by NIV.

### NIV induced mitochondrial ultra-structural changes

**Figure 5:**
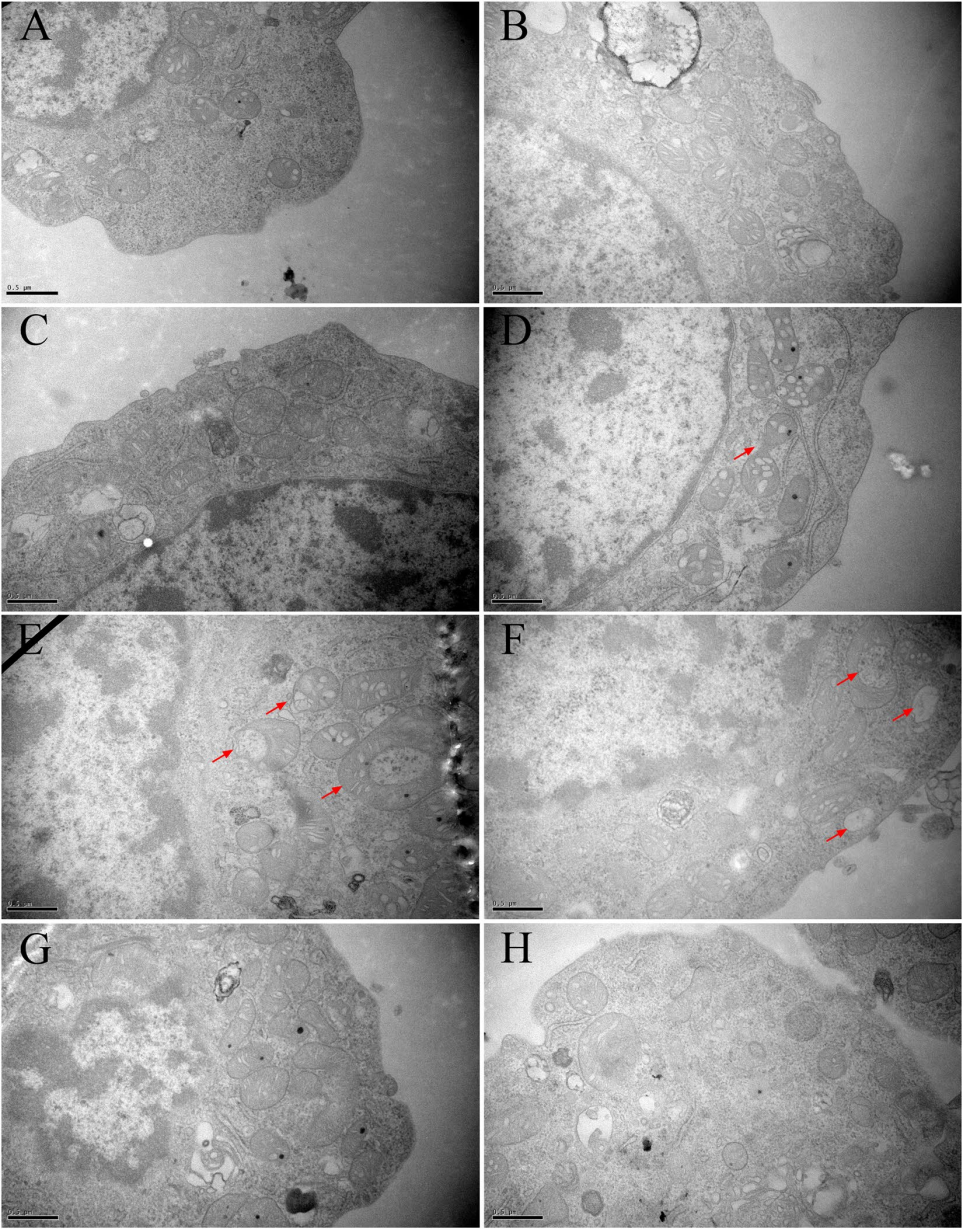
NIV-induced ultrastructural changes of mitochondria in GH3 cells (30000×). (**A**) control group; (**B**) Z-VAD-FMK group; (**C**) hemoglobin group; (**D**,**E**,**F**) 2 μM NIV-treated group, showing abnormal morphology and mitochondria swelling, absence of crista, vacuolization in mitochondria. (**G**) NIV and Z-VAD-FMK co-treatment group. (**H**) NIV and hemoglobin co-treatment group.

In the present study, several mitochondrial ultrastructural changes were observed in GH3 cells after NIV (2 μM) treatment (Fig. 5D,E and F). These mainly included abnormal morphology and swelling, absence of crista, vacuolization in mitochondria. However, when the cells were pretreated with Z-VAD-FMK (Fig. 5G) or hemoglobin (Fig. 5H) followed by NIV (2 μM) exposure, they presented almost normal mitochondrial morphology as the control (Fig. 5A). The cells treated with Z-VAD-FMK (Fig. 5B) or hemoglobin (Fig. 5C) alone showed normal mitochondrial morphology. These results further indicated that the caspase signaling pathway played an important role in the induction of mitochondrial dysfunction and apoptosis. NO could be the initiating agent to generate oxidative stress, target the mitochondria, activate the caspase signaling pathway and finally induce apoptosis.

### NIV changed the mRNA level of growth-associated genes in GH3 cells

**Figure 6:**
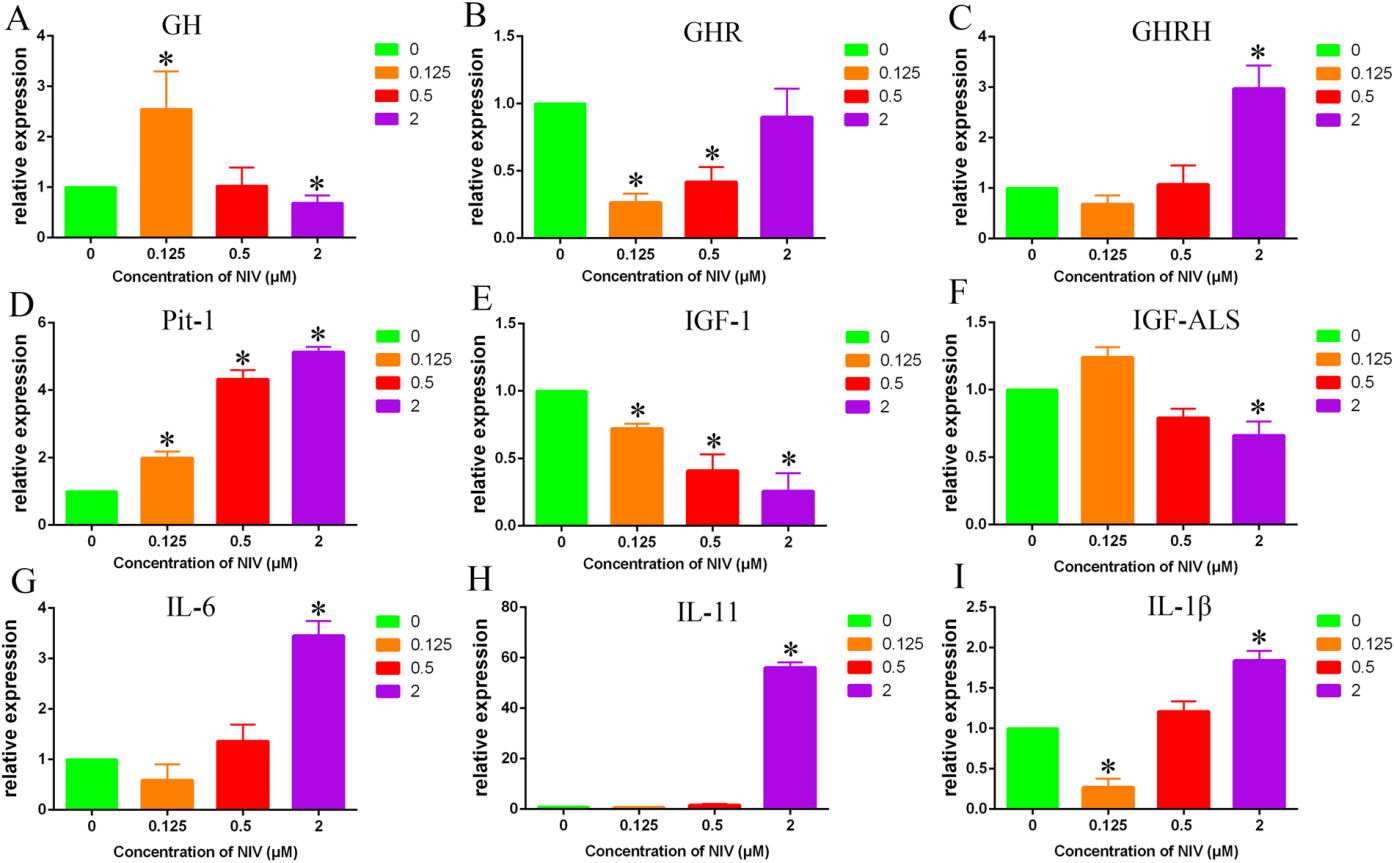
NIV-induced gene expression of growth-associated genes and cytokines in GH3 cells. The relative expression of GH (**A**), GHR (**B**), GHRH (**C**), Pit (**D**), IGF-1 (**E**), IGF-ALS (**F**), IL-6 (**G**), IL-11 (**H**), IL-1β (**I**) were assessed by qRT-PCR. The data was expressed as mean ± SD of 3 separate experiments performed in triplicate and were analyzed using a one-way ANOVA. *p < 0.05 denotes statistical significance versus control.

The growth-associated genes GH, GHR and growth hormone-releasing hormone (GHRH), pituitary-specific transcription factor-1 (Pit-1), IGF-1 and IGF-ALS were measured in GH3 treated with NIV. As shown in Fig. 6, the GH mRNA level was significantly increased at 0.125 μM NIV, but significantly decreased at 2 μM NIV (p < 0.05). However, the GH mRNA level remained unchanged after 0.5 μM NIV treatment (Fig. 6A). The GHR mRNA level was significantly decreased after 0.125 and 0.5 μM NIV treatment (p < 0.05). Interestingly, NIV at 2 μM did decrease GHR mRNA level, but with no statistical significance (p > 0.05) (Fig. 6B). NIV at 0.125 and 0.5 μM slightly decreased or increased the GHRH mRNA level (p > 0.05). However, 2 μM NIV increased the mRNA level of this gene (p < 0.05) (Fig. 6C). NIV at 0.125, 0.5 and 2 μM significantly increased the Pit-1 mRNA level (p < 0.05) (Fig. 6D), and decreased IGF-1 mRNA level (p < 0.05) (Fig. 6E) with a concentration-dependent manner. NIV at 0.125 μM increased IGF-ALS mRNA level, but decreased it at 0.5 and 2 μM NIV (Fig. 6F).

Since the previous study suggested that cytokines, IL-6, IL-11 and IL-1β may be involved in growth retardation by upregulating the expression of SOCSs, which are upstream inhibitors of IGF-ALS and IGF-1^9^. We measured the effects of NIV on the mRNA level of IL-6, IL-11 and IL-1β cytokines. NIV at 0.125 μM decreased the mRNA level of these genes. However, NIV at 0.5 and 2 μM increased the mRNA level of IL-6 (Fig. 6G), IL-11 (Fig. 6H) and IL-1β (Fig. 6I). Particularly, 2 μM NIV had a remarkable promoting effect on the gene expression of IL-11 (56.1-fold). These results indicated that NIV could indeed induce the gene expression of cytokines like IL-6, IL-11 and IL-1β.

### Inhibition of GH secretion in GH3 cells by NIV in NO-dependent manner

**Figure 7:**
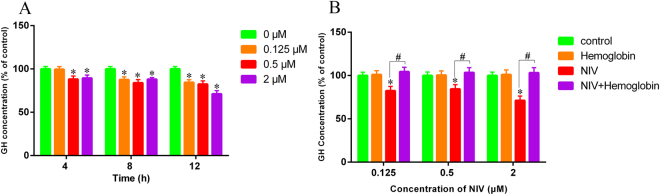
The GH level in the culture medium of GH3 cells after treatment of NIV without (**A**) or with (**B**) hemoglobin was measured. The data were expressed as mean ± SD of 6 separate experiments performed in triplicate. Comparisons between multiple groups with different concentrations of NIV (0.125, 0.5 and 2 μM) were analyzed using a one-way ANOVA. *p < 0.05 denotes statistical significance versus control. Comparisons between two groups with or without hemoglobin were analyzed using Student’s t-test. ^#^p < 0.05 denotes statistical significance versus NIV alone treated cells.

The GH level in the culture medium of GH3 cells after exposure to NIV with or without pretreatment of hemoglobin was measured. As shown in Fig. 7A, NIV decreased the GH level in a time- and concentration-dependent manner. NIV at 0.125 μM did not noticeably affect the GH level until 8 h of exposure. NIV at 0.5 and 2 μM significantly decreased the GH level after as early as 4 h of exposure, achieving the maximum effect after 12 h of exposure. However, pretreatment with hemoglobin completely reversed the inhibition of GH level induced by all concentrations of NIV (Fig. 7B), suggesting that NO played an upstream role in inhibition of GH secretion by NIV.

### Confirmatory experiments in primary rat pituitary cells

To confirm the toxic effects and mechanism of growth retardation caused by NIV, we replicated the most stunning experiments in isolated primary rat pituitary cells. The primary pituitary cells treated with given concentrations of NIV for 12 h showed obvious toxic changes according to the cell density, cell growth state and cell morphology when compared with the control group (see supplementary Figure 1). We measured the NO generation in the primary cultures after NIV incubation for 12 h, and the results showed that NIV could increase the NO levels in primary pituitary cells with a concentration-dependent manner (supplementary Figure 2A). To explore the mediating actions of NO in the *GH* gene expression and activation of caspase-3, -8 and -9, we pretreated the isolated primary cells with NO scavenger hemoglobin for 30 minutes followed by co-incubation of cells with NIV and hemoglobin for 12 h. the results showed that NIV indeed inhibited the *GH* gene expression at middle and high concentrations (0.5 and 2 μM), which however, is partially alleviated by hemoglobin (supplementary Figure 2B). NIV activated the caspase pathway by significantly increasing the mRNA levels of caspase-3 and -9 in primary pituitary cells, but not the mRNA levels of caspase-8. However, the hemoglobin partially reversed the activation of caspase-3 and -9 (supplementary Figure 3). These results suggested that NO played an upstream role in NIV-induced inhibition of *GH* gene expression and activation of caspase-3 and -9 in primary pituitary cells, which is consistent with the results in GH3 cells.

## Discussion

Nivalenol (NIV), a type B trichothecenes commonly found in cereal crops, has been reported to play negative impact on weight gain in mice^23,24^, rats^11,25^, chickens^26,27^ and pigs^28^. This effect was commonly referred to as growth retardation^9,29^. Growth retardation is a very unspecific outcome for trichothecenes, which may be due to the effects of these toxins on the central nervous system, reduced feed consumption or hormonal changes^5^. Initial studies suggested that trichothecene-induced weight reduction was due to decreased appetite (feed refusal) involving serotonin, which is supported by the apparent alteration of serotonin in the brain of animals^30,31^. However, following study failed to find out a strong correlation between weight reduction and feed refusal in DON-exposed animals at dietary concentrations of less than 25 ppm^32^. Previous studies demonstrated that T-2 toxin could induce a substantial effect of oxidative stress in the brain of mice^33–35^. Furthermore, the study of Sehata *et al*.^36^ showed that T-2 toxin increased the number of apoptotic neuroepithelial cells in the telencephalon of rat fetal brain and strongly induced oxidative stress-related genes. All these studies indicated that the brain is an important target organ for trichothecenes, which is probably related to growth retardation.

Limited but available data indicated that NIV could be distributed in multiple tissues including liver, kidney, spleen, bile, muscle, intestine, heart, brain and colon content in broiler chickens and piglets^37,38^. In broiler chickens, the circulating levels could reach 62.56 ± 30.86 ng/ml (equal to 0.2 μM) after oral administration of NIV at a dosage of 0.8 mg/kg b.w.^37^. The doses used in the present study (0.125, 0.5, 2 μM) is comparable to the circulating levels that could be achieved in animals, when considering that the average levels of NIV in maize and wheat samples for farm animals could reach 0.53, 1 and 15.28 mg/kg in Zimbabwe (in 2014)^2^, Brazil (in 2009)^1^ and Hubei of China (in 2009)^39^, respectively. Besides, the natural exposure of animals to NIV was a long process, which make the animals more prone to be influenced by the toxin. Therefore, the doses used here was referential.

As part of the brain, pituitary cells are responsible for producing hormones like GH. Earlier studies showed that the anterior pituitary was affected by NIV and DON^10,11^, yet an alteration in GH level was not reported. In addition, a mechanistic study of NIV-induced growth retardation involving toxicity of pituitary cells and GH suppression has not been systematically investigated. Here, we assumed that NIV-induced growth retardation might be due, on one hand, to the oxidative stress-mediated mitochondrial dysfunction and apoptosis of anterior pituitary cells, and on the other hand, to the changes in growth-associated gene expression and GH deficiency.

Oxidative stress in the form of ROS or RNS (reactive nitrogen species) generation or disruption of the redox homeostasis in the cells is not only involved in cell signaling and self-defense, but also induces apoptosis. It was indicated that trichothecenes increased the ROS level in the cells, which caused lipid peroxidation, DNA damage, disruption of redox homeostasis and apoptosis^40–42^. For RNS, previous studies suggested that T-2 toxin or DON could change the inducible NOS activity in human intestinal cells^43^, macrophage cells^14^ and in mice^44^ and rats^35^. However, there is no report that NIV changes the inducible NOS activity and increases the NO in pituitary cells. In the present study, we confirmed that NIV significantly increased the NO level, but not the ROS level, to mediate the oxidative stress. Furthermore, the NO generation was due to the increase in activity of endothelial NOS but not inducible NOS in pituitary GH3 cells. We found that NIV did not significantly induce ROS generation, which is paradoxical with previous study, where NIV (0.5–5 μM) induced a significant increase of ROS after 20 h exposure of intestinal epithelial cells to toxin^45^. The paradoxical results indicate that the type of cell lines, dose of toxin and exposure time may influence the ROS generation.

It was reported that T-2 toxin and DON stimulated lipid peroxidation and changed the activities of antioxidant enzymes in a variety of cells *in vitro*, and in chickens, mice and rats *in vivo* ^40,42,46,47^. However, little information is available on NIV-induced lipid peroxidation and alterations in the antioxidant system. In our research, we showed that NIV caused a significant increase in MDA level (an index of cellular lipid peroxidation) in GH3 cells. In addition, NIV induced a significant decrease in GSH level with a concentration-dependent manner. The activities of critical antioxidant enzymes like SOD, CAT and GSH-Px were also altered significantly.

NO, as a free radical, could induced lipid peroxidation and changed the activities of SOD, CAT and GSH-Px^48^. NO-mediated oxidative stress has been identified as an important underlying mechanism of apoptosis and mitochondrial dysfunction in human gingival fibroblast and osteoblast cells^16,49^. The fact that no obvious increase in ROS level was observed indicated that the increased MDA level and GSH depletion were possibly due to NO generation caused by NIV in GH3 cells.

Trichothecenes have been reported to induce apoptosis in various cells and the activation of caspase signaling pathway is fundamental to apoptotic process. For example, it was reported that T-2 toxin activated the caspase-dependent (intrinsic caspases-9 and -3 pathway) and independent AIF pathway to induce apoptosis in human cervical cancer cells, where oxidative stress was suggested to be an underlying mechanism^21^. DON induced oxidative stress and mitochondrial dysfunction in mice thymic epithelial cells, which might contribute to apoptosis. Furthermore, the caspase-3, -8 and -9 molecules were activated as well^50^. In the present study, we showed that NIV could induce apoptosis in GH3 cells. With regard to mitochondrial injuries, NIV at 0.5 and 2 μM significantly decreased the ΔΨm in cells. Furthermore, NIV obviously changed the morphology of the mitochondria as visualized by abnormal morphology and swelling, absence of crista and vacuolization in mitochondria. Downstream of the mitochondrial damage, NIV activated the caspase signaling pathway, including the caspase-9 molecule (specific to the intrinsic mitochondrial apoptotic pathway), the caspase-8 molecule (specific to the extrinsic pathway) and the execution caspase-3 molecule (molecule where the intrinsic and extrinsic pathways converge). These results suggested that NIV not only activated the mitochondrial apoptotic pathway through caspase-9, but also activated the extrinsic pathway through caspase-8. However, caspase inhibitor Z-VAD-FMK could partially protect cells from NIV-induced apoptosis and partially reversed the activation of caspase pathway. These findings suggested that NIV-induced apoptosis is caspase dependent and that other caspase-independent mechanisms of cell death might be involved. Importantly, the NO scavenger hemoglobin also reversed the activation of caspase pathway and decrease of ΔΨm and recovered the morphological changes of mitochondria caused by NIV. This suggested that NO-mediated oxidative stress might play an important role in NIV-induced mitochondrial dysfunction and apoptosis of GH3 cells.

Previous results from our laboratory found that both T-2 toxin and DON had an inhibitory effect on the *GH* gene expression and GH secretion in GH3 cells^12^. DON was reported to perturb the GH axis by suppressing two relevant growth-related proteins, IGF-1 and IGF-ALS, to induce growth retardation^9,51^. Pro-inflammatory cytokine expression was suggested to affect growth through the induction of several SOCSs in the mice exposed to DON^52^. In the present study, we showed that NIV at low concentration (0.125 μM) significantly increased the GH mRNA level, but significantly decreased it at high concentration (2 μM). Meanwhile, NIV at 0.5 and 2 μM significantly decreased the GH secretion as early as 4 h after toxin treatment. NIV at 0.125 μM decreased the GH secretion after 8 h of treatment. Alternatively, other growth-associated genes including GHR, GHRH, Pit-1, IGF-1 and IGF-ALS were also altered by NIV. These genes played important roles in production, release and regulation of signal transduction of GH. Specifically, NIV increased the mRNA level of GHRH at 0.5 and 2 μM and Pit-1 at all given concentrations. This seemed to be contradictory with the result that NIV decreased the GH secretion, because GHRH stimulates GH production and secretion by binding to the GHRH receptor in anterior pituitary cell and Pit-1 is a pituitary-specific transcription factor responsible for pituitary development and growth hormone expression in mammals^53,54^. Additionally, we showed that NIV could decrease the expression of IGF-1 and IGF-ALS and induce pro-inflammatory cytokines IL-6, IL-11 and IL-1β expression. We thought that the inhibition of GH secretion by NIV was a complex process. Importantly, our results also showed that the decrease of GH secretion caused by NIV was completely recovered by NO scavenger hemoglobin, suggesting that NO indeed played a critical role in NIV induced GH deficiency.

To assure the universality and repeatability, we replicated some of the most stunning results in isolated primary culture of pituitary cells from rat. We showed that NIV at given concentrations had an obvious toxic effects on primary cultures, which was manifested by decrease in cell density, change in cell growth state and cell morphology. In accordance with the results in GH3 cells, NIV at 0.5 and 2 μM significantly increased the NO generation with a concentration-dependent manner after 12 h of exposure. For the caspase pathway, NIV at all given concentrations significantly increased the mRNA levels of caspase-9, but not the caspase-8, which suggested that NIV primarily activated the intrinsic mitochondrial apoptotic pathway to mediate the apoptosis in primary cultures of pituitary cells. Downstream of the caspase-9, NIV at 2 μM significantly increased the mRNA levels of caspase-3. However, the NO scavenger hemoglobin reversed the activation of caspase pathway induced by NIV in primary cultures, which is consistent with that in GH3 cells. Furthermore, hemoglobin also partially reversed the inhibition of *GH* gene expression caused by NIV in primary cultures. These results indicated that NO was indeed the major contributor to the oxidative stress caused by NIV in not only the GH3 cells but also in the primary rat pituitary cells. Besides, NO played a universal role in NIV-induced caspase activation and GH deficiency in these two type of pituitary cells.

In conclusion, the present study demonstrated that NIV could cause obvious cytotoxicity, NO generation, oxidative stress, mitochondrial dysfunction and apoptosis in GH3 cells. The oxidative stress led to lipid peroxidation and GSH depletion and altered the redox homeostasis in cells. The NO, but not the ROS, was the major contributor of oxidative stress. Furthermore, NIV changed the expression of growth-associated genes like *GH*, *GHR*, *GHRH*, *Pit-1*, *IGF-1* and *IGF-ALS* and *IL-6*, *IL-11* and *IL-1β* cytokines, and decreased the GH secretion. Importantly, this study also found that NO played an upstream role in the activation of caspase-3, -8 and -9, mitochondrial injuries and GH secretion in GH3 cells. This study threw a new light on NIV-induced oxidative stress, apoptosis and growth hormone deficiency, which might provide a new target for the treatment of growth retardation caused by NIV.

## Materials and Methods

### Materials

NIV (CAS NO. 23282-20-4) was purchased from Sigma (Aldrich, Fluka). Dulbecco’s Modified Eagle’s Medium (DMEM) was obtained from Hyclone (Logan, USA). Fetal bovine serum (FBS), antibiotics (penicillin, streptomycin) and trypsin-EDTA solution were supplied by Gibco-BRL Life Technologies (Logan, UT). NIV was dissolved in dimethyl sulfoxide (Amresco, USA). 3-(4, 5-dimethylthiazol-2-yl)-2, 5-diphenyltetrazolium bromide (MTT) was obtained from Biotech (USA). 2′,7′-dichlorodihydrofluorescein diacetate (DCFH-DA), 3-amino, 4-aminomethyl-2′,7′-difluorescein diacetate (DAF-FM DA), Rhodamine 123 (Rh 123), NAC and SOD (65 KU/mg) were purchased from Sigma Chemicals Co. (St Louis, Missouri, USA). An Annexin V-PI apoptosis kit was obtained from BestBio. The LDH, SOD, GSH-Px, CAT assay kits were acquired from Jiancheng-Bioengineering Institute (Nanjing, P.R. China). The cleaved-caspase-3 (Asp175) was purchased from Cell Signaling Technology (U. S. A), and a reagent Western Blot Stripping Buffer (T7135A) were purchased from Takara (Japan). The MDA assay kit and β-actin antibody (AA128) and caspase inhibitor Z-VAD-FMK were obtained from Beyotime Institute of Biotechnology (Nantong, P.R. China). A rat growth hormone (GH) ELISA kit was from Merck Millipore Corporation.

### Cell culture

GH3 cells (Cell Bank of Academy of Sciences, Shanghai, P.R. China) were cultured in DMEM supplemented with 10% heat-inactivated fetal bovine serum and 1% penicillin-streptomycin at 37 °C in a humidified atmosphere of 5% CO_2_ in air. After 24 h, the culture medium was replaced by DMEM containing 1% heat-inactivated FBS and 1% penicillin-streptomycin and incubated with or without NIV at indicated time intervals. GH3 cells passaged 5–15 times were used in the experiments. All experiments were performed at least in triplicate on three separate occasions.

### MTT, LDH leakage and apoptosis assays

Cytotoxicity was assayed by MTT and LDH leakage assays. For the MTT assay, GH3 cells were seeded in 96-well culture plates (1 × 10^3^ cells/well) and were cultured for 24 h. Then the cells were treated with NIV (0.125, 0.5, 2 and 5 μM) at 37 °C for 4, 8, 12, and 24 h, respectively. After incubation, the cells were treated with 0.5 mg/ml MTT (100 μl/well) at 37 °C for 4 h. and then the optical density was read using a microplate reader (BioTek Instruments, Inc., Winooski, VT, USA). The IC50 of NIV to GH3 cells was calculated using SPSS 13.0 (regression-probit analysis) for Windows.

For the LDH leakage assay, GH3 cells were seeded in 6-well culture plates (5 × 10^5^ cells/well) and were cultured for 24 h, and then treated with 0.125, 0.5 and 2 μM of NIV at 37 °C for 12 h. The LDH release in the cell supernatant was measured using a commercial kit (Nanjing Jiancheng Bioengineering Institute, Nanjing, P.R. China). The absorption was measured at 450 nm using a microplate reader as described above.

Cell apoptosis assays were performed using an Annexin V-PI apoptosis detection kit, as described in our earlier study^52^. Briefly, GH3 cells were seeded in 6-well culture plates (5 × 10^5^ cells/well) and were cultured for 24 h, and then treated with 0.125, 0.5 and 2 μM of NIV at 37 °C for 12 h. After treatment, the cells were washed twice with phosphate-buffered saline (PBS) and incubated in 300 μl binding buffer containing 3 μl Annexin V-FITC and 3 μl propidium iodide at room temperature in the dark for 15 minutes. Flow cytometric analysis of apoptosis was performed with a Beckman Coulter CyAn ADP flow cytometer (Beckman, California, USA) following the manufacturer’s protocol.

### Measurement of NO and nitric-oxide synthase

Determination of intracellular NO was based on the oxidation of 3-amino, 4-aminomethyl-2′,7′-difluorescein diacetate (DAF-FM DA), resulting in the formation of the fluorescent compound 3-amino, 4-aminomethyl-2′, 7′-difluorescein (DAF-FM). Briefly, GH3 cells (5 × 10^5^ cells/well) were seeded in black 6-well plates and were cultured for 24 h at 37 °C. Then the cells were treated with 0.125, 0.5 and 2 μM NIV for 12 h followed by fluorescent probe DAF-FM DA (final concentration is 5 μM) incubation for 30 minutes at 37 °C. Then the cells were washed three times and the dynamic changes of fluorescence intensity was determined within 2 h using Tecan infinite F200 microplate reader (TECAN, Austria) at an excitation wavelength of 495 nm and an emission wavelength of 515 nm. For hemoglobin pretreatment, the cells (5 × 10^5^ cells/well) were seeded in black 6-well plates and were cultured at 37 °C for 24 h. Then the cells were pretreated with hemoglobin (20 μM) for 30 minutes and then exposed to NIV for 12 h followed by DAF-FM DA treatment for 30 minutes. Then the cells were washed and the fluorescence intensity was determined as described before.

NO is synthesized through NOS with L-arginine and molecular oxygen as substrates. Determination of NOS was based on the oxidation of DAF-FM DA by NO when the substrate L-arginine is abundant. The specific inducible NOS inhibitor SMT and endothelial NOS inhibitor L-NAME (Beyotime Inst. Biotech, Peking, P.R. China) were used to demonstrate which NOS is involved in NIV-induced NO production. Briefly, GH3 cells (1 × 10^3^ cells/well) were seeded in black 96-well plates and were cultured for 24 h at 37 °C. Then the cells were treated with 0.125, 0.5 and 2 μM NIV for 12 h followed by fluorescent probe DAF-FM DA incubation for 30 minutes at 37 °C. Afterwards, 100 μl SMT (1 mM) or 100 μl L-NAME (100 μM) and 100 μl NOS detection solution (Beyotime Inst. Biotech, Peking, P.R. China) containing L-arginine, 0.1 mM NADPH, 5 μM DAF-FM DA and NOS detection buffer were added and incubated for 40 minutes at 37 °C. Then the cells were washed three times and the fluorescence intensity was determined using Tecan infinite F200 microplate reader (TECAN, Austria) as described before. All the assays were carried out at least in triplicate on three separate occasions.

### Determination of lipid peroxidation and antioxidant enzymes

Lipid peroxidation was determined by measuring MDA using a commercial kit (Beyotime Inst. Biotech, Peking, P.R. China). GSH, SOD, CAT and GSH-Px activities were determined using commercial kits (Nanjing Jiancheng Bioengineering Institute, Nanjing, P.R. China). Briefly, GH3 cells (5 × 10^5^ cells/well) were seeded in 6-well plates and were cultured for 24 h at 37 °C. Then the cells were treated with 0.125, 0.5 and 2 μM NIV for 12 h. then the cells were collected, washed and lysed with radio immuno precipitation assay (RIPA) lysis buffer (Beyotime Inst. Biotech, Peking, P.R. China) containing 50 mM Tris, 150 mM NaCl, 1% Triton-X-100, 1% sodium deoxycholate, 1% SDS, 0.1 mM EDTA and 1 mM phenyl methyl sulfonyl fluoride (pH 7.4). The cell lysates were further disrupted using an ultrasonication instrument (CV18, Xinchen, P.R. China) in ice and then centrifuged at 1600 rpm for 10 minutes. The supernatant was collected for the determination of MDA, GSH, SOD, CAT and GSH-Px. The detailed procedures followed the manufacturer’s instructions.

Intracellular ROS formation was measured by using an oxidation sensitive fluorescent probe, DCFH-DA. Briefly, GH3 cells were seeded in black 6-well culture plates (5 × 10^5^ cells/well) and were cultured for 24 h at 37 °C, Then the cells were treated with 0.125, 0.5 and 2 μM NIV for 12 h followed by incubation with 1 ml DMEM containing DCFH-DA (final concentration is 10 μM) for 30 minutes at 37 °C. The cells were washed, harvested with trypsin-EDTA and resuspended with PBS. The dynamic changes of fluorescence intensity of ROS within 2 h were measured using Tecan infinite F200 microplate reader (TECAN, Austria) at an excitation wavelength of 488 nm and an emission wavelength of 525 nm.

### Measurement of ΔΨm

Loss of ΔΨm was assessed by using a fluorescent indicator Rhodamine 123 (Rh 123). Rh 123 is cell membrane permeable and accumulates in mitochondria with active membrane potential and fluorescence increases due to dye stacking and decreases when agents disrupt ΔΨm. Briefly, GH3 cells were seeded in 12-well culture plates (1 × 10^4^ cells/well) and were cultured at 37 °C for 24 h. Afterwards, the cells were treated with NIV for 12 h and were harvested, washed twice with ice-cold PBS and then were resuspended in DMEM containing Rh123 (final concentration is 2 μM) for 30 minutes at 37 °C in the dark. The cells were then washed with ice-cold PBS three times. Flow cytometric analysis of ΔΨm was performed with a Beckman Coulter CyAn ADP flow cytometer (Beckman, California, USA) following the manufacturer’s protocol. For pretreatment of SOD (final concentration is 200 U/ml), NAC (final concentration is 1 mM) and hemoglobin (final concentration is 20 μM). GH3 cells were pretreated with these agents for 30 minutes, then were incubated with NIV for 12 h. The subsequent procedures were done as described before.

### Morphological and ultrastructural studies: transmission electron microscopy (TEM)

GH3 cells were seeded in a 75 cm^2^ flask (5 × 10^5^ cells/ml) and were cultured to about 80% confluence. The cells were treated with 2 μM NIV for 12 h, and then were fixed in 2.5% (v/v) glutaraldehyde. Cell precipitation was prepared by centrifugation at 800 rpm for 5 minutes and then placed at room temperature for at least 4 h. The cells were washed 3 times in distilled water and post-fixed for 2 h in 1% osmium tetroxide (w/v). The cells were washed again 3 times, dehydrated stepwise in ethanol, and then embedded by polymerization at 45 °C and 60 °C for 12 h and 36–48 h, respectively. Ultra-thin sections (70 nm) were stained with lead citrate for 10 minutes and uranyl acetate for 30 minutes, then rinsed with distilled water 3 times and dried. The sections were viewed on an H-7650 TEM (Hitachi, Japan).

### Total mRNA isolation, reverse transcription and quantitative real-time PCR

The mRNA level of caspase-3, -8 and -9, GH, GHR, GHRH, Pit-1, IGF-1, IGF-ALS, IL-6, IL-11 and IL-1β were determined by quantitative real-time PCR (qRT-PCR) as previously described by Wang *et al*.^55^. Briefly, GH3 cells were seeded in 6-well culture plates (5 × 10^5^ cells/well) and were cultured at 37 °C for 24 h. The cells were then treated with NIV for 12 h. For pretreatment of Z-VAD-FMK, the cells were pretreated with Z-VAD-FMK (final concentration is 20 μM) for 30 minutes and then were treated with NIV for 12 h. Total RNA was isolated from the cells using TRIzol reagent (Takara, Dalian, P.R. China) according to the manufacturer’s instructions. One microgram of total RNA was reverse-transcribed to complementary DNA (cDNA) with the use of a ReverTra Ace First Strand cDNA Synthesis Kit (Promega). cDNA was amplified by qRT-PCR (BioRad, Hercules, CA) using a SYBR Premix Ex Taq RT-PCR kit (Takara, Code QPK-201, P.R. China). Each 25 μl reaction mixture was consisted of 12.5 μl SYBR Premix Ex Taq, 0.5 μl of each primer (10 μM), 2 μl of cDNA and 9.5 μl RNase-free dH_2_O. Cycling conditions were as follows: step 1, 30 s at 95 °C; step 2, 40 cycles at 95 °C for 5 s, 60 °C for 30 s; step 3, 95 °C for 15 s, 60 °C for 30 s, 95 °C for 15 s. Data from the reaction was collected and analyzed by complementary computer software. Relative quantification of gene expression was calculated using the 2^−ΔΔCt^ method, and normalized to β-actin in each sample. The primers used in this study were shown in Table 1.

**Table 1:** Primers used for qRT-PCR analysis.

Genes	Sequence (5′-3′)	Amplified region (bp)
β-actin	S: 5′ GCAGGAGTACGATGAGTCCG 3′	74
	A: 5′ ACGCAGCTCAGTAACAGTCC 3′	
GH	S: 5′ CTTCTCGCTGCTGCTCATC 3′	214
	A: 5′ GTTGGCGTCAAACTTGTCATA 3′	
GHR	S: 5′ AGACTGGATAAAGAGCACGAA 3′	147
	A: 5′ CTGGGAATTGAACTTGGGT 3′	
GHRH	S: 5′ CAGGACGGAGAAGGAGG 3′	203
	A: 5′ GGTGAGGAGCACAAAGAAC 3′	
Pit-1	S: 5′ CAACGTGATGTCCACAGCGA 3′	77
	A: 5′ CGTAGGTGGATGGCTGGTTT 3′	
IGF-1	S: 5′ GCACCTCCAATAAAGATACAC 3′	137
	A: 5′ AACTGAAGAGCGTCCACC 3′	
IGF-ALS	S: 5′ TACCAACCGCCTCACACATC 3′	103
	A: 5′ CTCCGCGGATAAAGTCGTCA 3′	
IL-1β	S: 5′ TGTGATGTTCCCATTAGAC 3′	131
	A: 5′ AATACCACTTGTTGGCTTA 3′	
IL-11	S: 5′ GCTTCCTGGAGTGCTGACAA 3′	155
	A: 5′ GTAAGCGACGAAGTAGCCGT 3′	
IL-6	S: 5′ CTGGTCTTCTGGAGTTCCGT 3′	147
	A: 5′ TCTTGGTCCTTAGCCACTCCT 3′	
Caspase-3	S: 5′ CTGGACTGCGGTATTGAG 3′	102
	A: 5′ GGGTGCGGTAGAGTAAGC 3′	
Caspase-8	S: 5′ AGGAAGCCTCTATCTATG 3′	152
	A: 5′ CTGTTCTAAGCCTGTCTC 3′	
Caspase-9	S: 5′ CCACTGCCTCATCATCAAC 3′	148
	A: 5′ CTTAGCGGTCAGGTCGTT 3′	

*Note:* The primers were manufactured by Nanjing Genescript Co. Ltd. (Nanjing, P.R. China). GH, growth hormone; GHR, growth hormone receptor; GHRH, growth hormone releasing hormone; Pit-1, pituitary-specific transcription factor-1; IGF-1, insulin-like growth factor-1; IGF-ALS, insulin-like growth factor acid-labile substance; IL-1β, interleukine-1 beta; IL-11, interleukin-11; IL-6, interleukin-6.

### Protein extraction and western blot

GH3 cells were seeded in 6-well culture plates (5 × 10^5^ cells/well) and were cultured at 37 °C for 24 h. The cells were then treated with NIV for 12 h. For pretreatment of Z-VAD-FMK, the cells were pretreated with Z-VAD-FMK (final concentration is 20 μM) for 30 minutes and then were co-incubated with NIV for 12 h. After incubation, the cells were harvested and washed with ice-cold PBS three times and lysed in RIPA lysis buffer containing protease inhibitor (1 mM of PMSF) (Sigma). The cell lysates were further disrupted using an ultrasonication instrument (CV18, Xinchen, P.R. China) in ice twice (5 s/time) and were placed in ice for 1 h. The lysates were centrifuged at 12000 rpm for 15 minutes at 4 °C and supernatants were subjected to western blot analysis. Protein (50 μg) was loaded for 1-dimensional SDS-PAGE using 12% separating gel and 5% stacking gel. The separated proteins were electrophoretically transferred to a polyvinylidene fluoride (PVDF) (Minipore) membranes in Trans-Blot Cells (Liuyi, Beijing, P.R. China). The membranes were blocked with 5% nonfat milk in Tris-buffered saline containing 0.1% Tween-20 (TBS-T) for 1 h and were then immunoblotted with the primary antibody (anti-caspase 3 antibody at a dilution of 1:2000) overnight at 4 °C. The membranes were washed for 10 minutes three times with TBS-T and then were incubated with horseradish peroxidase-conjugated secondary anti-IgG antibody (diluted 1:5000) (Beyotime Inst. Biotech, Peking, P.R. China) at room temperature for 1 h. The membranes were washed for 10 minutes three times with TBS-T. Immunoreactive bands were visualized with a chemiluminescent substrate (ECL-Plus, Minipore) for 2–5 minutes. Images were captured with a LAS-4000 luminescent image analyzer (Fujifilm, Tokyo, Japan). The photographs generated were quantitatively analyzed with a Quantity One image densitometer. Protein level were standardized by comparison with β-actin.

### Measurement of growth hormone by ELISA

GH3 cells were seeded in 24-well culture plates (1 × 10^4^ cells/well) and were cultured for 24 h. The cells were treated with NIV for 4, 8 and 12 h. For pretreatment of hemoglobin, the cells were pretreated with hemoglobin for 45 minutes, and then were co-incubated with NIV for 12 h. After incubation, the medium were collected and centrifuged at 2000 rpm at 4 °C for 5 minutes. The supernatant were collected for measurement of growth hormone using a rat growth hormone ELISA kit (Merck Millipore Corp.), according to the manufacturer’s instructions.

### Isolation and primary culture of pituitary cells from rat

The isolation and primary culture of pituitary cells from rat were performed according to previous studies^56,57^ with a little modification. In brief, the adolescent female rat was anaesthetized and the anterior pituitary was removed immediately after decapitation and placed in sterile 0.3% BSA-CMF hanks buffer at 4 °C. The pituitary was then washed with ice-cold 0.3% BSA-CMF hanks buffer, chopped in approximately 1 mm^3^ pieces with a scalpel and washed again. The tissue fragments were treated with trypsin-EDTA solution for 30 minutes at 37 °C. The trypsin-EDTA was then removed and the tissue fragments were washed and treated with collagenase type II for 30 minutes at 37 °C. Subsequently, the cell suspension was filtered through a nylon mesh and centrifuged for 5 minutes at 1000 rpm. Cells were then resuspended in DMEM complete medium (HyClone, USA) supplemented with 20% fetal bovine serum and seeded at a density of 4–5 × 10^5^ cells/cm^2^ in 25 mm culture flask or 24-well plate (Corning, USA) coated with poly-l-lysine. The cells were kept at 37 °C in a humidified atmosphere of 5% CO2 in air until start of experiments. The cell morphology was observed under inverted microscope (OLYMPUS, Japan).

### Statistics

Statistical difference of all data was performed using SPSS 13.0 for Windows. All results were presented as mean ± standard deviation (SD). Differences between two groups in the case of experiments with or without Z-VAD-FMK or hemoglobin were assessed by Student’s t-test. Differences between multiple groups in all figures were analyzed using a one-way analysis of variance (ANOVA). A statistically significant difference was indicated when the p-value < 0.05.

## Electronic supplementary material

Supplementary Information
