# Supplementary material for: Nitric oxide mediates apoptosis and mitochondrial dysfunction and plays a role in growth hormone deficiency by nivalenol in GH3 cells
Source: Sci Rep. 2017 Dec 6;7:17079. doi: 10.1038/s41598-017-16908-y (PMC5719085; doi:10.1038/s41598-017-16908-y)
Supplement: Supplementary file 1 — Supplementary Information [file 41598_2017_16908_MOESM1_ESM.pdf]

Title page:

Nitric oxide mediates apoptosis and mitochondrial dysfunction and plays a role in growth hormone deficiency by nivalenol in GH3 cells

Deyu Huang (The first author),

The Key Laboratory for the Detection of Veterinary Drug Residues,

Ministry of Agriculture, P.R. China

LuQing Cui

Laboratory of Quality & Safety Risk Assessment for Livestock and Poultry Products (Wuhan), Ministry of Agriculture, P.R China

Pu Guo

Laboratory of Quality & Safety Risk Assessment for Livestock and Poultry Products (Wuhan), Ministry of Agriculture, P.R China

Xijuan Xue

Laboratory of Quality & Safety Risk Assessment for Livestock and Poultry Products (Wuhan), Ministry of Agriculture, P.R China

Qinghua Wu

College of Life Science, Yangtze University, Jingzhou, Hubei, 434025, P.R. China

Hafiz Iftikhar Hussain

Laboratory of Quality & Safety Risk Assessment for Livestock and Poultry Products (Wuhan), Ministry of Agriculture, P.R China

Xu Wang (co-corresponding author)

Laboratory of Quality & Safety Risk Assessment for Livestock and Poultry Products (Wuhan), Ministry of Agriculture, P.R China

Tel: 0086-27-87287186-317; Fax: 0086-27-87672232;

E-mail address: wangxu@mail.hzau.edu.cn

Zonghui Yuan (corresponding author)

The Key Laboratory for the Detection of Veterinary Drug Residues,

Ministry of Agriculture, P.R. China

Tel: 0086-27-87287186; Fax: 0086-27-87672232;

E-mail address: yuan5802@mail.hzau.edu.cn

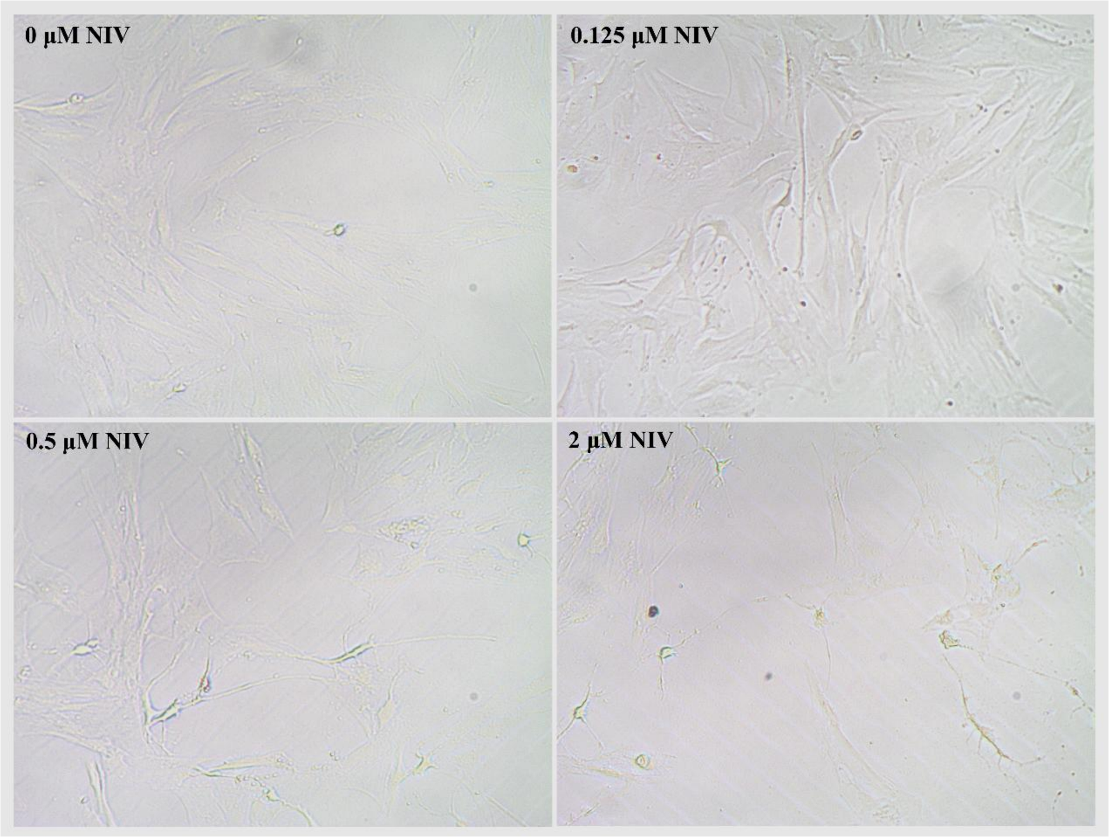

Supplementary figure 1. The observation of cell morphology of primary cultures of rat pituitary cells after 0.125, 0.5 and 2 µM NIV treatment (220 ×).

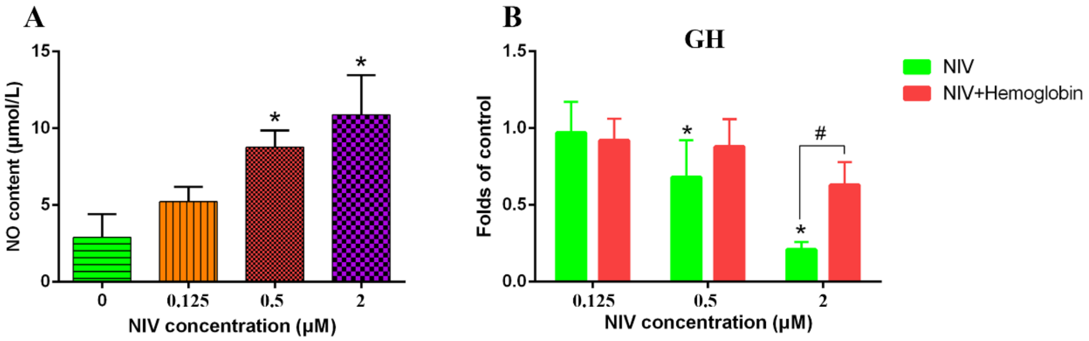

Supplementary figure 2. NIV induced NO generation (A) and inhibited the gene expression of *GH* gene (B) in primary cultures of rat pituitary cells. The NO content in primary cultures after NIV treatment for 12 h was measured using a commercial total nitric oxide assay kit (Beyotime Inst. Biotech, Peking, P.R. China). The relative expression of *GH* gene was assessed by qRT-PCR. The

results were expressed as mean ± SD of 3 separate experiments performed in triplicate and were statistically analyzed using one-way ANOVA. * $p<0.05$ denotes statistical significance versus control. Comparisons between two groups without or with hemoglobin were analyzed using Student's t-test. # $p<0.05$ denotes statistical significance versus NIV alone treated cells.

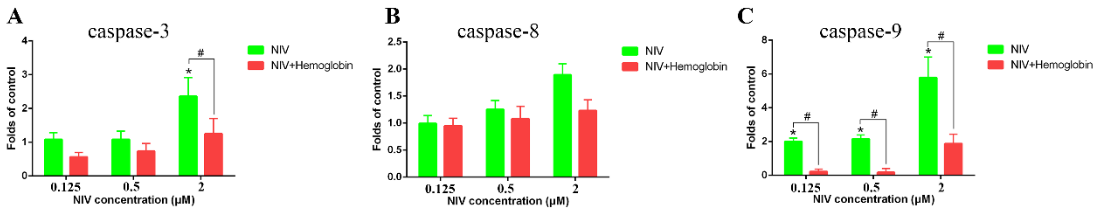

Supplementary figure 3. NIV-induced caspase activation in primary cultures of rat pituitary cells. The gene expression of caspase-3 (A), caspase-8 (B) and caspase-9 (C) induced by NIV without or with hemoglobin was assessed by qRT-PCR. The data was expressed as mean ± SD of 3 separate experiments performed in triplicate. Comparisons between multiple groups with different concentrations of NIV (0.125, 0.5 and 2 μM) were analyzed using a one-way ANOVA. * $p<0.05$ denotes statistical significance versus control. Comparisons between two groups without or with hemoglobin were analyzed using Student's t-test. # $p<0.05$ denotes statistical significance versus NIV alone treated cells.
